# The correlation between iodine and metabolism: a review

**DOI:** 10.3389/fnut.2024.1346452

**Published:** 2024-03-19

**Authors:** Le Zhang, Fangjian Shang, Cong Liu, Xiaodan Zhai

**Affiliations:** ^1^Department of Endocrinology, Shengjing Hospital of China Medical University, Shenyang, China; ^2^Department of General Surgery, The Fourth Affiliated Hospital of China Medical University, Shenyang, China

**Keywords:** iodine, metabolism, obesity, dyslipidemia, antioxidant

## Abstract

Iodine is involved in the synthesis of thyroid hormones and plays a crucial role in human life. Both iodine deficiency and excess are common issues in certain populations. Iodine also has extrathyroidal effects on organs that can uptake it independently of thyroid hormones. Recently, multiple clinical studies have shown a connection between iodine intake and metabolic disorders, such as metabolic syndrome, obesity, diabetes, hypertension, and dyslipidemia. However, the results of these studies have been inconsistent, and the mechanisms behind these associations are still not well understood. Therefore, in this review, we aim to examine the recent research progress regarding the relationship between iodine and metabolic disorders, along with the relevant mechanisms.

## 1 Introduction

Iodine is an elementary micronutrient for human life. It exist in various forms in human body, such as iodide atom (I^−^), molecular iodine (I_2_), triiodide (I_3_), iodine anion (HI_2_O^−^), and iodine-binding molecules (such as iodolipids) (1). I^−^ is a kind of reducing agent that can be oxidized by peroxidase enzymes to generate thyroid hormones (THs). Tri-iodothyronine (T3) and thyroxine (T4) are essential regulators of energetic metabolism (1). Recent studies have indicated that iodine, aside from its role in the thyroid, also functions as an antioxidant, immunomodulator, and differentiator in various organs and tissues (2).

Metabolic syndrome (MetS) is defined as the combination of metabolic disorders including abdominal obesity, hypertension (HBP), dyslipidemia, and hyperglycemia (3). MetS is widely prevalent worldwide and poses serious issues such as cardiovascular disease (CVD), tumors, and total mortality (4). The development of MetS involves multiple factors, with research indicating that oxidative stress and chronic inflammatory conditions play a vital role (5). Changes in dietary habits are another contributing aspect to the prevalence of MetS. Consequently, factors associated with MetS, such as iodine nutritional status, may partially explain its occurrence of MetS.

In this review, we focus on iodine and its connection to metabolic disorders, as well as the associated mechanisms. The findings will aid us in acquiring a comprehensive understanding of iodine’s role and provide supporting evidence for an appropriate and secure iodine nutrition standard.

## 2 The molecular basis of extrathyroidal effects of iodine

Sodium iodide symporter (NIS) is the most effective and specific transporter of I^−^ expressed on thyroid follicular epithelial cells. Many other organs can also actively accumulate iodine, including the salivary glands, stomach, lactating mammary gland, ovary, prostate, and pancreas. In addition to NIS and Pendrin, new iodine transporters, including cystic fibrosis transmembrane conductance regulator (CFTR), anoctamin 1 (ANO1), and sodium multivitamin transporter (SMVT), have recently been discovered to be expressed in these organs (2). Therefore, iodine itself has extrathyroidal effects on organs that can take it up.

## 3 Sources and safety concentration of iodine

In order to eliminate iodine deficiency diseases, most countries have implemented universal salt iodization (USI). However, excess iodine intakes can occur due to the consumption of iodized salt, drinking water, animal milk rich in iodine, specific types of seaweeds, iodine-containing dietary supplements, and from a combination of these sources. Some pharmaceuticals (like Amiodarone), disinfectants, and iodine-containing contrast media, can also be common sources of iodine (6). Over 90 percent of dietary iodine is absorbed under normal conditions (7). The recommended dietary intake of iodine is 150–299 μg/day (8, 9). The appropriate range of median urinary iodine concentration (mUIC) should be 100–299 μg/L. Japanese average iodine consumption is 1,200 μg/day, which is 7.2 times higher than that of the British and 5.7 times higher than that of Americans (10). Iodine excess can increase the risk of hyperthyroidism and subclinical hypothyroidism (11). However, euthyroid individuals are usually tolerant to iodine-induced thyroid diseases as their thyroid function rapidly normalizes upon discontinuation of excessive iodine consumption (8, 12). A moderately high iodine intake has been shown to be beneficial in reducing the incidence of breast and prostate cancer in some epidemiological studies (13–15).

## 4 Clinical research on the correlation between iodine status and metabolic disorders

Recent research has examined the impact of iodine itself on the prevalence of metabolic disorders. The majority of clinical studies were published within the past 5 years. Various indicators were used to evaluate iodine nutritional status, including urinary iodine concentration (UIC), water iodine concentration (WIC), and daily iodine consumption. However, due to the use of different indicators for assessing iodine nutrition, varying diagnostic criteria for metabolic disorders, and differences in the age and gender of the subjects, the studies did not yield consistent findings. The representative studies on the relationship between iodine and metabolic disorders have been summarized in Table 1.

**Table 1:** Summary of the correlation between iodine status and metabolic diseases.

No.	Region, study	Author, year	Study design	Iodine status groups	Subjects	The characteristics of thyroid function	Main finding(s)
1	America, NHANES III	Inoue K, et al., 2018 (16)	Longitudinal	UIC (<50, 50–99, 100–299 [Ref], 300–399, and ≥ 400 μg/L)	12,264 adults	Undescribed	Excessive iodine (UIC ≥400 μg/L) increased the risk of all-cause mortality (HR =1.19, 95% CI 1.04–1.37).
2	Spain, Di@bet.es	Maldonado-Araque C, et al., 2021 (17)	Longitudinal	UIC (<50, 50–100, 100–300 [Ref], and ≥ 300 μg/L)	4,370 adults	With difference	Iodine deficiency (UIC <50 μg/L) increased the risk of all-cause mortality (HR =1.71, 95% CI 1.18–2.48).
3	France, E3N-EPIC	Mancini FR, et al., 2019 (18)	Longitudinal	Dietary iodine intake (29.3–116.9 [Ref], 117.0–138.9, 139.0–160.7, 160.7–190.5, and 190.6–596.8 μg/d)	71,264 women	With difference	High iodine intakes (160.7–190.5 and 190.6–596.8 μg/d) were associated with a higher risk of developing T2DM (HR =1.27, 95% CI 1.10–1.47 and HR =1.28, 95% CI 1.07–1.53).
4	Korea, MRCohort	Park JK, et al., 2021 (19)	Longitudinal	Dietary iodine intake and seaweed consumption	2,588 postmenopausal women	Undescribed	Average iodine and seaweed consumption was inversely associated with MetS incidence and its individual abnormalities.
5	Belgium, ENVIRONAGE birth cohort	Neven KY, et al., 2021 (20)	Longitudinal	Placental iodine	471 mother-neonate pairs	Undescribed	A higher concentration of iodine in the placenta was associated with a reduced incidence of GDM (OR = 0.82; 95%CI 0.72–0.93).
6	America, NHANES 2007–12	LEE K W, et al., 2016 (21)	Cross-sectional	UIC (UIC below vs. above the 10th percentile)	2,495 adults	Undescribed	Low UIC was associated with higher TC (OR = 1.51, 95% CI = 1.03–2.23) and LDL-C (OR = 1.58, 95% CI = 1.11–2.23), and lower HDL/LDL ratio (<0.4) (OR = 1.66, 95% CI = 1.18–2.33).
7	America, NHANES 2001–12	Wang X, et al., 2019 (22)	Cross-sectional	UIC (low = UIC < 49 μg/L and normal = UIC ≥ 49 μg/L)	1,692 adolescents	Undescribed	Low UIC was associated with elevated TC (95% CI 1.37–2.81), elevated non-HDL (95% CI 1.33–2.76) and elevated LDL (95% CI 1.83–4.19).
8	China	Liu J, et al., 2019 (23)	Cross-sectional	Iodine-adequate area (mUIC 126.6 μg/L); Iodine-sufficient area (mUIC 221.2 μg/L); Iodine-excess area (mUIC 421.3 μg/L)	825 adults	With difference	Blood glucose, as well as systolic and diastolic pressure of adults in both iodine-sufficient and iodine-excess areas were higher (all *p* < 0.001).
9	China, TIDE	Jin M, et al., 2020 (24)	Cross-sectional	UIC (<100, 100–299 [Ref], 300–499, 500–799, and ≥ 800 μg/L)	51,795 adults	Without difference	The association between UIC and the prevalence of various metabolic disorders was U-shaped.
10	China, TIDE	Lu X, et al., 2020 (25)	Cross-sectional	UIC (<100, 100–199 [Ref], 200–299, and ≥ 300 μg/L)	75,653 adults	Undescribed	Subjects in the IS and IE groups had a lower probability of having hyperuricemia and gout in comparison to those in the IA group
11	China	Zhao J, et al., 2021 (26)	Cross-sectional	UIC (<100, 100–199 [Ref], and ≥ 200 μg/L)	2,691 adults	Without difference	UIC was inversely associated with the prevalence of MetS.
12	America, NHANES 2011–12	Ezemaduka Okoli CB, et al., 2021 (27)	Cross-sectional	UIC (low = UIC < 100 μg/L and normal = UIC ≥ 100 μg/L)	1,286 adults	Undescribed	Low UIC was associated with higher FPG (OR = 1.73, 95% CI = 1.09–2.72) in females.
13	China	Wang D, et al., 2021 (28)	Cross-sectional	Iodine-deficient area (mWIC <10 μg/L); iodine-adequate area (mWIC 40–100 μg/L); Iodine-excess area (mWIC >100 μg/L);	1,235 adults	Undescribed	Iodine-excess was a protective factor against high TG (OR = 0.649, 95% CI 0.455–0.924) and low HDL-C (OR = 0.429, 95% CI 0.264–0.697).
14	China	Wang D, et al., 2021 (29)	Cross-sectional	Iodine-adequate area (mWIC 71.4 μg/L); Iodine-excess area (mWIC 325 μg/L)	144 pregnant women, 237 lactating women, 828 adults	With difference	There was a positive correlation between systolic pressure and WIC, while the blood glucose level showed an inverse association with both WIC and UIC.
15	America	Villatoro-Santos CR, et al., 2022 (30)	Cross-sectional	24 h urinary iodine excretion (≥300 μg/d) and concentration (≥300 μg/L)	217 school-age children and 478 parents	Undescribed	High UIC was associated with MetS, but not high 24 h urinary iodine excretion.

UIC, urinary iodine concentration; WIC, water iodine concentration; BMI, body mass index; TSH, thyrotropin; TC, total cholesterol; TG, triglyceride; HDL-C, high-density lipoprotein cholesterol; LDL-C, low-density lipoprotein cholesterol; DM, diabetes mellitus; IFG, impaired fasting glucose; IGT, impaired glucose tolerance; WC, waist circumference; MetS, metabolic syndrome; HR, hazard ratio; CI, confidence interval; AOR, adjusted odds ratio; HOMA-IR, homeostatic model assessment of insulin resistance; HbA1c, glycated hemoglobin; FPG, fasting plasma glucose.

### 4.1 Iodine and metabolic syndrome

In cross-sectional studies, the relationship between UIC and MetS prevalence was found to be inversely associated or exhibited a U-shaped curve, with the lowest point observed at a UIC of 300–499 μg/L (24, 26). A prospective study indicated that dietary iodine and seaweed consumption was inversely associated with MetS incidence in Korean postmenopausal women (19). Furthermore, high seaweed intake was negatively associated with the incidence of MetS in men with the TG and TT genotypes of lipoprotein lipase gene (LPL) rs17482735 (31), However, another cross-sectional study conducted on school-age children and their parents revealed that high UIC was associated with MetS (30).

### 4.2 Iodine and obesity

In a large epidemiological study (TIDE) conducted in China, the prevalence of central obesity significantly decreased when the UIC was 300 μg/L or higher. The odds ratio (OR) for central obesity with an UIC of ≥800 μg/L was 0.797 (*p* < 0.05) (24). Among school-age children in China, overweight children exhibited a lower UIC compared to children with normal weight (32). Women with obesity also demonstrated a significantly lower UIC in comparison to both themselves after undergoing bariatric surgery and women with normal weight (33). In a randomized controlled trial (RCT), the body fat percentage of the participants who consumed tablets containing iodine-reduced kelp powder showed a significant decrease in comparison to those who took the placebo (34). In a 4-week placebo-controlled study, seaweed fucoxanthin supplementation (1 mg/day) decreased waist circumference (WC) and fat mass in obese Japanese individuals. In addition, fucoxanthin supplementation (3 mg/day) decreased visceral fat, body mass index (BMI), and weight (35). However, mUIC was positively associated with obesity among Colombian women of reproductive age (36).

### 4.3 Iodine and hyperglycemia

The association between UIC and the prevalence of diabetes exhibited a U-shaped curve in the TIDE study (24). In a cohort study involving 71,264 women, individuals with higher levels of iodine intake were found to be at a higher risk of developing type 2 diabetes mellitus (T2DM) in comparison to those with inadequate iodine intake (18). Adults in iodine-sufficient (IS, mUIC 200–299.99 μg/L) and iodine-excess (IE, mUIC ≥300 μg/L) areas exhibited higher blood glucose levels compared to those in iodine-adequate area (IA, mUIC 100–199.99 μg/L) (23). Low UIC was also reported to be associated with an increased risk of elevated fasting plasma glucose (FPG) in females (27). A study revealed that patients with T2DM had lower UIC levels compared to healthy individuals. Furthermore, UIC showed a negative correlation with insulin resistance in subjects with T2DM (37). However, in a 4-week clinical trial, seaweed supplementation (48 g/day) decreased blood glucose levels in Korean patients with T2DM (38).

Higher iodine status may potentially protect against hyperglycemia during pregnancy. A study indicated that pregnant and lactating women in the IE area (mWIC >300 μg/L) had lower blood glucose levels and a lower prevalence of hyperglycemia (29). Furthermore, a higher concentration of iodine in the placenta was associated with a reduced incidence of gestational diabetes mellitus (GDM) among 471 pregnant women (20). However, Bell et al. did not find any correlation between UIC and the prevalence of GDM (39).

### 4.4 Iodine and hypertension

In the TIDE study, researchers observed a U-shaped curve in the relationship between UIC and the prevalence of hypertension. The lowest point was found at a UIC range of 300–499 μg/L (24). Two studies conducted in China revealed that adults in IS and IE areas exhibited higher blood pressure levels compared to those in IA area (23, 29). Meanwhile, iodine deficiency was identified as a risk factor for preeclampsia and hypertensive disease of pregnancy (HPD) (40). However, a randomized case–control study revealed that wakame (Undaria pinnatifida) intake (5 g/day) in brown algae significantly decreased blood pressure in 36 older Japanese individuals with hypertension (41).

### 4.5 Iodine and dyslipidemia

The National Health and Nutrition Examination Survey (NHANES) 2007–2012 reported that subjects with the lowest decile of UIC were more likely to be at risk for elevated total cholesterol (TC) (adjusted odds ratio (aOR) = 1.51) and elevated low-density lipoprotein (LDL) cholesterol (aOR = 1.58), compared to those with the highest decile of UIC (21). US adolescents with low UIC had a significantly higher risk of hypercholesterolemia, elevated non-high-density lipoprotein (HDL), and elevated LDL compared to those with normal UIC (22). RCTs have reported that iodine supplementation reduces hypercholesterolemia incidence in overweight women (42) and also decreased serum LDL-C levels in overweight Japanese adults (34). Seaweed supplementation increased HDL-C levels and decreased TG levels in Korean patients with T2DM (38). A meta-analysis found that brown seaweed intake significantly decreased the levels of TC (mean difference (MD): −3.001; 95% CI: −5.770, −0.232) and LDL-C (MD: −6.519; 95% CI: −12.884, −0.154) (43). However, there is a observational study that propose the opposite conclusion (23). Two studies have found that the relationship between iodine status and dyslipidemia is either a U-shaped (24) or inverted U-shaped curve (28).

### 4.6 Iodine and hyperuricemia and gout

So far, the relationship has only been reported in one epidemiological study, which found an inverse association between UIC and the prevalence of hyperuricemia and gout. Subjects in the IS and IE groups had a lower probability of having hyperuricemia and gout in comparison to those in the IA group (25).

### 4.7 Iodine and mortality risk

Longitudinal data indicated an excess mortality in individuals with ID (UIC <100 μg/L) after adjusting for confounding factors. The HRs for all-cause mortality were 1.29 in individuals with UIC of 50–99 μg/L, and 1.71 in individuals with UIC of less than 50 μg/L. Iodine excess did not increase the risk of mortality (17). However, the NHANES III reported contradictory outcomes. In a median follow-up period lasting 19.2 years, having a UIC higher than 400 μg/L indicated an increased risk (HR = 1.19) for all-cause mortality. There was no observed correlation between low UIC and an increased risk of mortality (16).

## 5 Basic research on the correlation between iodine and metabolism

Clinical studies have demonstrated that iodine nutrition has an impact on metabolism. However, the mechanism involved in these effects is still poorly understood and requires further investigation. Due to varying dosages of iodine administration, durations of intervention, experimental animals, and thyroid hormone values, different *in vivo* studies have failed to reach a consensus. Kroupova et al. discovered that iodine intake could result in a dose-dependent elevation in blood cholesterol levels among hens (44). A study demonstrated that iodine excess (2.4 and 4.8 mg/L) could induce hepatic steatosis in BaLB/c mice, in a dose-dependent manner (45). However, higher iodine intake was found to benefit lipid metabolism in mice without significant differences observed in thyroid hormone levels and body weights among different groups (46). Iodine deficiency increased fat contribution to energy expenditure through elevated thyrotropin (TSH) in male mice (47). The metabolomics study of the repeated intervention of potassium iodide (KI) on adult male rats indicated a metabolic shift in the thyroid. This shift was also observed in the plasma and urine, and the metabolites were involved in pathways of metabolic regulators, branched-chain amino acids, oxidant stress, and inflammation-associated response (48).

## 6 The extrathyroidal mechanisms of iodine

Iodine is a micronutrient that possesses antimicrobial properties. Iodine treatment in obese mice showed a weight-reducing effect and modified the gut microbiota, leading to an increase in pathogenic bacteria and a decrease in beneficial bacteria. Conversely, contrasting response patterns were observed in mice with normal weight (49). Another study found a significant relationship between the use of vulvar povidone iodine disinfection and the colonization of neonatal oral microbiota (50). Additionally, the intestinal microbiota also contributed to iodine absorption (51). Therefore, it is feasible that iodine has an impact on metabolism by altering the microbiota.

There is considerable evidence indicating that iodine has extrathyroidal effects as an antioxidant, especially in breast diseases and certain tumors (14). Iodide has been found to be highly efficient in scavenging reactive oxygen species (ROS), thus reducing damage caused by free oxygen radicals (52). In lactating women, the iodine content in breast milk exhibits a negative correlation with the activity of catalase, superoxide dismutase (SOD), and glutathione peroxidase (GSH-Px), as well as adiponectin levels (53). As an obesity-related hormone, adiponectin also plays a crucial role in regulating insulin sensitivity. Seaweed supplementation increased antioxidant enzyme activities in a clinical trial of Korean patients with T2DM (38). Administering an iodide supplement between 100 and 300 μg/d increased the total antioxidant status in human serum (54). However, a recently published study showed that excessive iodine levels lead to cell growth inhibition, oxidative stress, and cellular apoptosis in pancreatic beta cells (55). By regulating oxidative status, iodine is associated with changes in insulin sensitivity or metabolism.

Chronic inflammatory condition paves the way for the development of metabolic disorders. Iodine also has well-known anti-inflammatory and immunomodulatory effects. Both PENDRIN and NIS were expressed on the surface of human leukocytes. The application of sodium iodide (NaI) to leukocytes resulted in a significant rise in the production of both pro- and anti-inflammatory cytokines (56). Administering an iodide supplement had a slight effect on the plasma concentration of inflammation markers and acute-phase proteins (54). Orally administered potassium iodide (15 mg/kg/day for 3 days) significantly inhibited the neutrophil chemotaxis in peripheral blood (57). Fernando et al. provided a summary of the current understanding regarding the potential anti-inflammatory properties of marine algae derivatives (58). They have been shown to reduce inflammation by targeting various cellular mechanisms, such as inhibiting pro-inflammatory enzymes like cyclooxygenase-2 (COX-2) and inducible nitric oxide synthase (iNOS), modulating mitogen-activated protein kinase (MAPK) pathways, and blocking nuclear factor kappa B (NF-κB) activation (59). MAPK pathway controls cellular growth processes and mitoses. Additionally, it is crucial for insulin resistance (60). NF-κB signaling is particularly relevant in inflammation-related diseases, including metabolic disorders. Therefore, it is plausible that iodine could have an impact on metabolism by modulating chronic inflammation.

**Figure 1:**
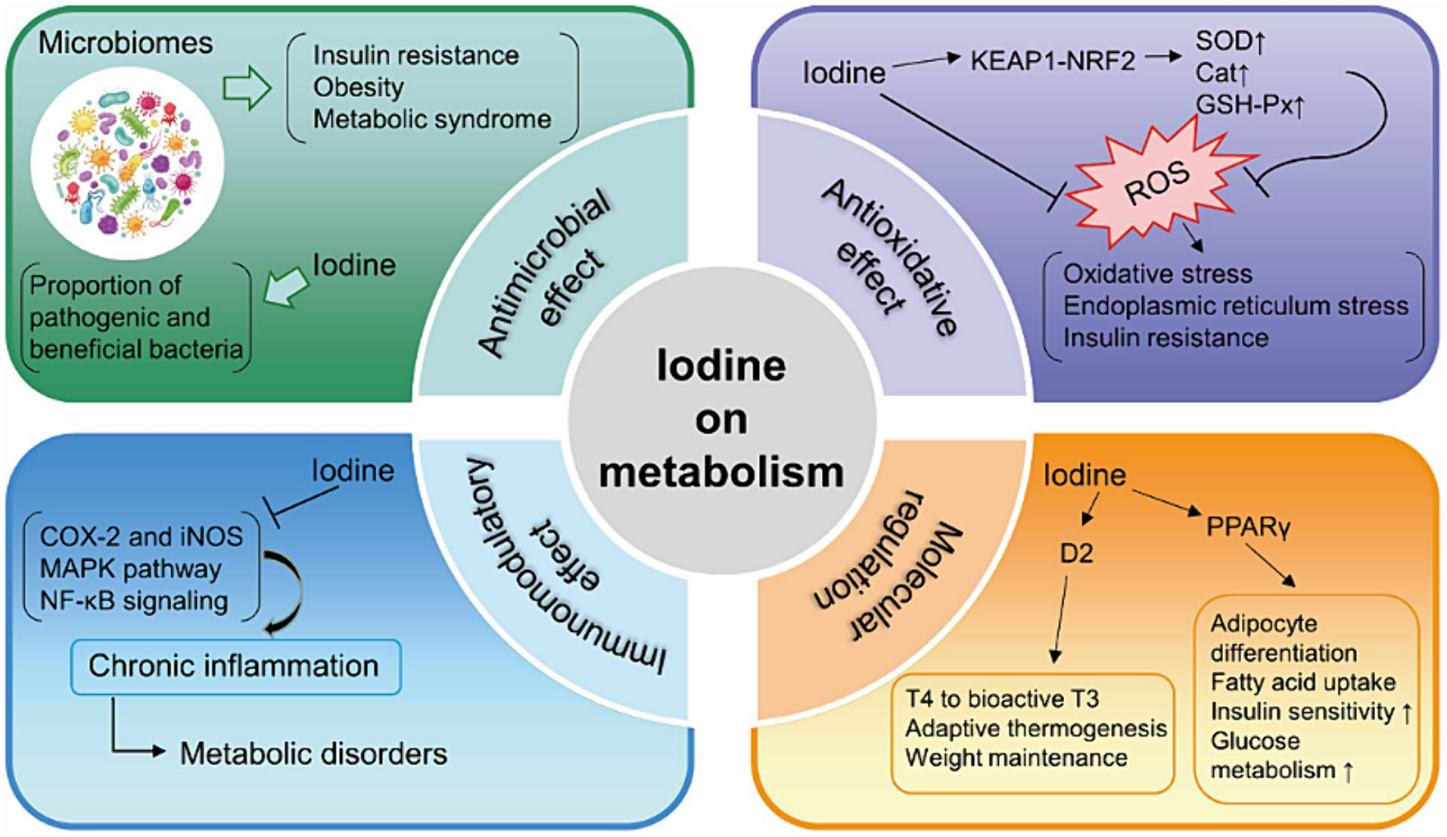
Summary of the mechanisms of iodine on metabolism. KEAP1, Kelch-like ECH-associated protein 1; NRF2, NF-E2-related factor 2; SOD, superoxide dismutase; Cat, catalase; GSH-Px, glutathione peroxidase; ROS, reactive oxygen species; COX-2, cyclooxygenase-2; iNOS, inducible nitric oxide synthase; MAPK, modulating mitogen-activated protein kinase; NF-κB, nuclear factor kappa B; PPARγ, peroxisome proliferator activated receptor-γ; D2, type 2 deiodinase; T4, thyroxine; T3, tri-iodothyronine.

I_2_ exhibited antiproliferative and apoptotic effects in mammary cancer models (61) through generating iodine-containing lipids (6-IL) and increasing peroxisome proliferator activated receptor-γ (PPARγ) expression (62, 63). PPARγ, expressed primarily in adipose tissue, promotes the differentiation of adipocytes, uptake of fatty acids, storage of triglycerides in lipid droplets. It increases insulin sensitivity and glucose metabolism (64). PPARα, PPARβ/δ and PPARγ are the three identified isoforms of PPARs. Table 2 presents the summarized relevant studies on the connection between iodine and PPARs. Additionally, excess iodine administration considerably hindered the activity of type 2 deiodinase (D2) in various organs, such as the pituitary, liver, and kidney (71–73). D2 is responsible for converting T4 to bioactive T3, which in turn promotes adaptive thermogenesis and is involved in weight maintenance (74). Figure 1 provides an overview of the potential mechanisms of iodine on metabolism.

**Table 2:** The correlation between iodine and PPARs.

Iodine and PPARs	Object	Mechanisms	Year, references
PPARγ agonist (rosiglitazone) → RAI uptake↑	Thyroid carcinoma patients	PPARγ expression increased in thyroid tissue	2008, (65)
I_2_↑ → PPARγ↑ I_2_↑ → PPARα↓	Human breast cancer cell line MCF-7	I_2_ treatment generates 6-IL derivative of AA, 6-IL binds specifically and with high affinity to PPARs	2009, (62)
I_2_↑ → PPARγ↑ I_2_↑ → PPARα↓	MUN induced mammary tumors in rats	The presence of AA and formation of its 6-IL derivative in tumoral mammary gland	2009, (66)
I_2_↑or I^−^↑ → PPARγ↑	DMBA induced mammary cancer in rats	Prevent estrogen-induced DNA adducts through PPARγ/caspases pathways	2011, (67)
Iodine deprivation→PPARγ↑	Trophoblastic Cells	Snail↑; MMP-9↑; GCM-1↓; hGC↓; PAPP-A↓; E-cadherin↓;	2016, (68)
I_2_↑ → PPARγ↑	HeLa and SiHa cervical cancer cells; NOD/SCID mice	CD49f, CK17, OCT-4, NANOG, SOX2 and KLF4↓	2018, (69)
I_2_↑ → PPARγ↑	Women with early (stage II) and advanced (stage III) breast cancer		2019, (70)

PPARs, peroxisome proliferator-activated receptors; AA, arachidonic acid; 6-IL, 6-iodo-5-hydroxy-eicosatrienoic acid and the 6-iodolactone; RXRs, retinoic X receptors; RAI, radioiodine; MNU, methyl-nitrosourea; DMBA, dimethylbenz [a] anthracene; ROS, reactive oxygen species; MMP-9, matrix metalloproteinase-9; GCM-1, glial cell missing-1; PAPP-A, pregnancy-associated plasma protein-A.

## 7 Summary

This review validates that iodine has effects on glucose metabolism, lipid metabolism, and obesity. The influence of iodine may be attributed to its antioxidant and immunomodulatory properties. Although the connections between iodine and metabolism are inconsistent, both iodine deficiency and prolonged iodine excess may pose a risk to thyroid disorders. It is important to maintain population iodine status within an optimal range. Further prospective studies and research on mechanisms are needed to establish an evidence-based and safe standard for iodine nutrition.

## Author contributions

LZ: Writing – original draft. FS: Writing – original draft. CL: Writing – review & editing. XZ: Writing – review & editing.
